# Comparison of epidural space contrast flow and clinical outcomes in parasagittal versus transforaminal epidural steroid injection

**DOI:** 10.1038/s41598-026-36056-6

**Published:** 2026-02-03

**Authors:** Narges Khojasteh, Hossein Majedi, Ali Emami Meibodi, Alireza Khajehnasiri, Reza Atef Yekta, Nader Ali Nazemian Yazdi, Mojgan Rahimi, Sogol Alikarami, Koorosh Kamali, Hamed Abdollahi

**Affiliations:** 1 Anesthesia, Critical Care, and Pain Management Research Center, Tehran University of Medical Sciences, Tehran, Iran; 2 Critical Care Medicine Department of Anesthesiology, School of Medicine, Besat Hospital, Hamadan University of Medical Sciences, Hamedan, Iran; 3 Pain Research Center, Neuroscience Institute, Imam Khomeini Hospital Complex, Tehran University of Medical Sciences, Tehran, Iran; 4 Department of Anesthesiology, Critical Care, and Pain, Dr. Ali Shariati Hospital, Tehran University of Medical Sciences, Tehran, Iran; 5 Department of Anesthesiology, Amir Alam Hospital Complex, Tehran University of Medical Sciences, Tehran, Iran; 6 Department of Public Health, School of Public Health, Zanjan University of Medical Sciences, Zanjan, Iran; 7 Imam Khomeini Hospital Complex, Keshavarz Blvd, Tehran, 1419733141 Iran

**Keywords:** Lumbosacral radicular pain, Parasagittal interlaminar epidural steroid injection, Transforaminal epidural steroid injection, Anterior epidural space, Neuropathic pain, Pain, Chronic pain, Neuropathic pain

## Abstract

Epidural steroid injections (ESI) are frequently used to treat lumbosacral radicular pain, but the solute spread in the epidural space needs further investigation. This semi-blind, randomized study assessed clinical outcomes and contrast spread patterns between the parasagittal interlaminar (PIL) and transforaminal (TF) approaches in 79 adults with low back pain. Participants were randomly assigned to receive either TF-ESI (3 ml) or high-volume PIL-ESI (10 ml). All procedures were performed under fluoroscopic guidance. Contrast spread was evaluated by a blinded pain specialist, and clinical outcomes, including analgesia, patient satisfaction, and quality of life, were measured at two weeks, one month, two months, and six months post-treatment. Results showed no differences in baseline characteristics between groups. There were no statistically significant differences between the two groups in mean pain intensity at baseline and six months after treatment (*p* = 0.590 and 0.484, respectively). Pain relief, satisfaction, quality of life, and contrast spread to the anterior epidural space did not differ over six months. However, the TF group required significantly more fluoroscopic images (*p* < 0.001). High-volume PIL-ESI provides clinical efficacy and anterior contrast distribution equivalent to TF-ESI, with fewer fluoroscopic images needed.

## Introduction

Lumbosacral radicular pain is a type of neuropathic pain that results from stimulation of the sensory root or dorsal root ganglion of the spinal nerve. It is caused by the production of abnormal impulses in the dorsal root ganglion and transmission through peripheral nerve axons. Treatment of radicular symptoms varies from lifestyle changes and conservative treatments to surgical interventions. Inadequate pain management is highly prevalent and associated with high costs and socioeconomic problems; therefore, attaining effective treatments is an important goal. Common interventional modalities for lumbosacral radicular pain include epidural steroid injection (ESI) and surgery^1–4^.

Epidural steroids show neuroprotective effects that attenuate nerve damage, inflammation, and edema, making ESI the most frequent intervention for controlling radicular symptoms^2,3^. However, the reported efficacy of ESI varies widely from 20% to 95%, depending on the injection method^1,2^. Some authors suggest that poor treatment outcomes are due to the failure of the injectate to reach the site of pathology in different injection techniques^5^. ESI can be performed by interlaminar (IL), transforaminal (TF), and caudal approaches. The IL approach can be performed using parasagittal (PIL) or midline (MIL) interlaminar techniques. In the IL method, the drug is primarily injected into the posterior epidural space, while in the TF method, the medication is placed near the nerve roots. In the PIL technique, unlike the usual midline approach, the drug is injected in the outermost part of the interlaminar space^6^.

A double-blind, randomized trial involving 37 patients with chronic lumbosacral radicular pain demonstrated that both TF and PIL techniques provided significant and comparable pain relief and functional improvement over a 6-month follow-up period. No significant differences were observed between these two groups in pain intensity, disability scores, or responder rates^7^. Comparable outcomes between TF and PIL methods have also been reported in several other studies^8–12^. A systematic review and meta-analysis comparing MIL, PIL, and TF-ESI techniques revealed that although short-term (1-month) pain relief was similar across PIL and TF, the PIL approach yielded significantly greater long-term pain reduction at 3 and 6 months. However, these findings were limited by high heterogeneity among the included studies^13^.

The American Society of Interventional Pain Physicians (ASIPP) classifies lumbar TF epidural injections below L4–5 as intermediate-risk procedures for bleeding, whereas lumbar IL injections are categorized as high-risk^14^. While the IL route carries a higher risk of dural puncture and unintentional intrathecal administration, the TF technique is more frequently associated with vascular entry and disc injury^15–18^. Although radiation exposure varies based on operator experience, equipment, and patient-related factors, several studies have noted lower radiation doses and reduced fluoroscopy times with the PIL method^8,11,19^. Moreover, PIL may provide superior relief for bilateral and multilevel symptoms or central canal stenosis, attributable to the injectate’s distribution within the dorsal epidural space^20^.

In the context of intervertebral disc disease, inflammatory reactions typically occur in the anterior epidural space as well as near nerve roots. Therefore, the optimal target for therapeutic delivery is likely the anterior or anterolateral epidural space between the disc and the nerve roots. Although neither procedure delivers medication precisely into this space, the TF approach is generally favored over IL methods due to its proximity to the ventral epidural space and presumed superior diffusion, theoretically allowing for smaller injectate volumes. However, this premise has not been definitively validated. Previous research indicates that with MIL injections, the injectate reaches the anterior epidural space in only 36% of cases, leading many physicians to prefer the TF method over the MIL technique^21^. Notably, some trials have observed better anterior contrast spread with the PIL approach compared to TF^11,19^, while others have found comparable ventral distribution between the two approaches^7,8^.

Few studies have evaluated the long-term effects of TF versus PIL-ESI^7,11,12^ or anterior contrast spread in these two techniques^7,8,11,19^. Most comparative trials have focused on pain scores or functional outcomes without correlating these endpoints with objective fluoroscopic or CT evidence of ventral distribution. Therefore, in this randomized clinical trial, we investigated anterior epidural contrast spread under fluoroscopic guidance and compared clinical efficacy and quality of life (QOL) improvement between the TF and PIL approaches over a 6-month follow-up. Specifically, we aimed to determine whether a high-volume PIL approach could achieve ventral targeting and analgesia comparable to the TF technique, thereby offering a potentially safer alternative for lumbosacral radicular pain.

## Materials and methods

### Study design and participants

The present study is a semi-blind, randomized clinical trial conducted at a tertiary university hospital. The study involves two parallel experimental arms: the transforaminal (TF) group and the parasagittal interlaminar (PIL) group. The protocol was approved by the Ethics Committee of Tehran University of Medical Sciences (ID IR.TUMS.IKHC.REC. 1397.359) and registered in the Iranian Registry of Clinical Trials (IRCT20200209046437N1) on 23/04/2020. All procedures were performed following the relevant guidelines and regulations. Written informed consent was obtained from each participant.

### Inclusion and exclusion criteria

Patients aged 20 to 80 years with unilateral lumbosacral radicular pain and MRI-confirmed lumbar disc pathology, including bulging, protrusion, or extrusion, with or without spinal canal or foraminal stenosis, enrolled in this study. Patients with a history of previous spine surgery, ESI into the lumbar EP space in recent years, allergy to medications, simultaneous topical steroid use, diabetic nephropathy, serum creatinine level > 2 mg/dl, opioid abuse, pregnancy, failed back surgery syndrome, and refusal to participate were excluded.

### Imaging review

All patients underwent MRI, and findings such as bulging, protrusion, and extrusion were documented. In cases where there was no significant canal and foraminal stenosis or degeneration, patients were categorized as having disc pathology without stenosis or degeneration.

### Radiographic assessment

**Fig. 1:**
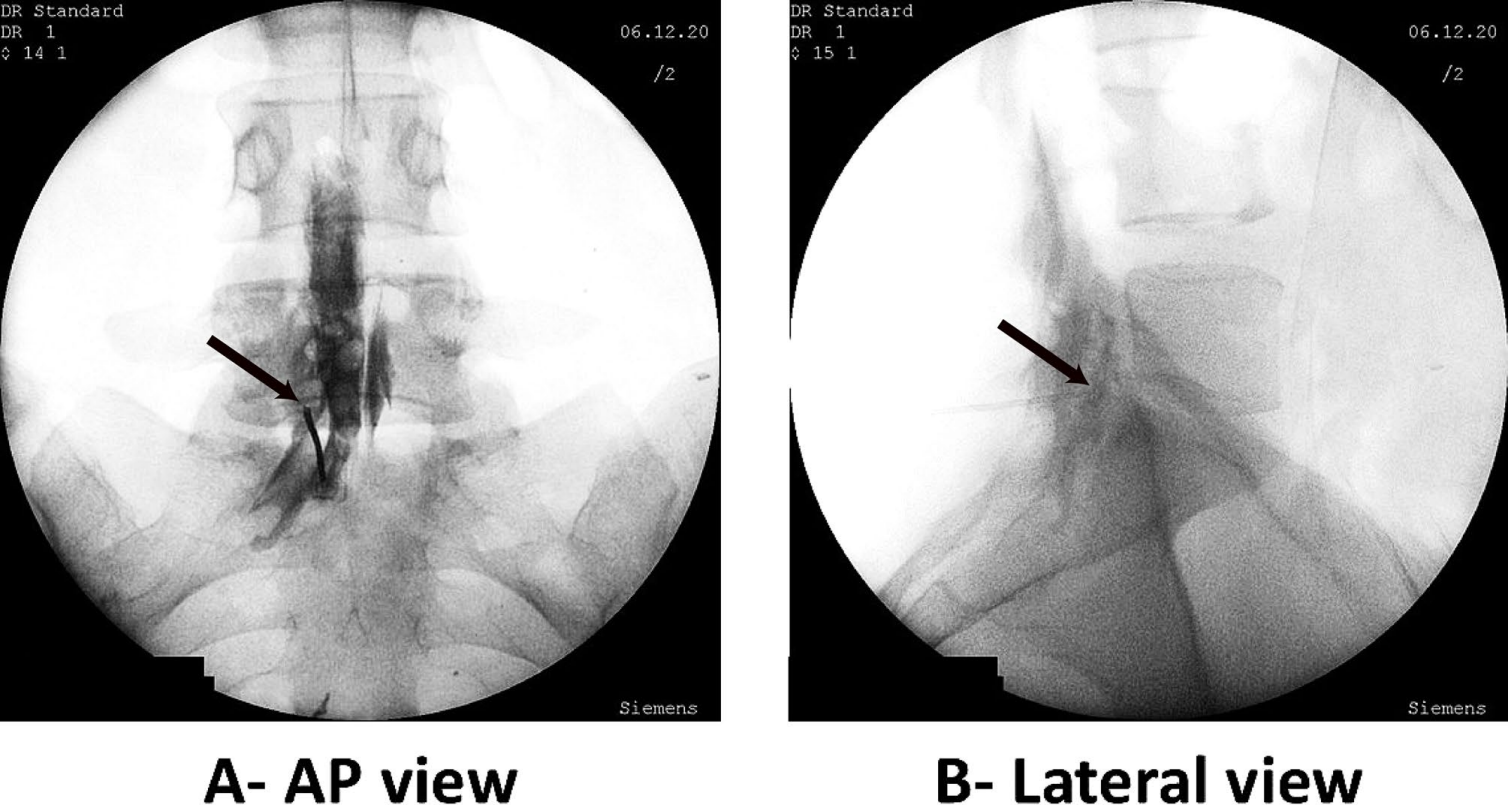
Black arrows indicate the needle tip position. The needle is placed in the left parasagittal L5-S1 epidural space (**A**). The contrast has spread to the anterior and posterior epidural spaces and adjacent segments (**B**). Cephalad and posterior spread in epidural space are more prominent.

A pain specialist, distinct from the treating physician and blinded to the group allocation, reviewed the fluoroscopic images and recorded the diffusion of the contrast agent into the anterior and posterior epidural space and adjacent segments (Fig. 1).

**Fig. 2:**
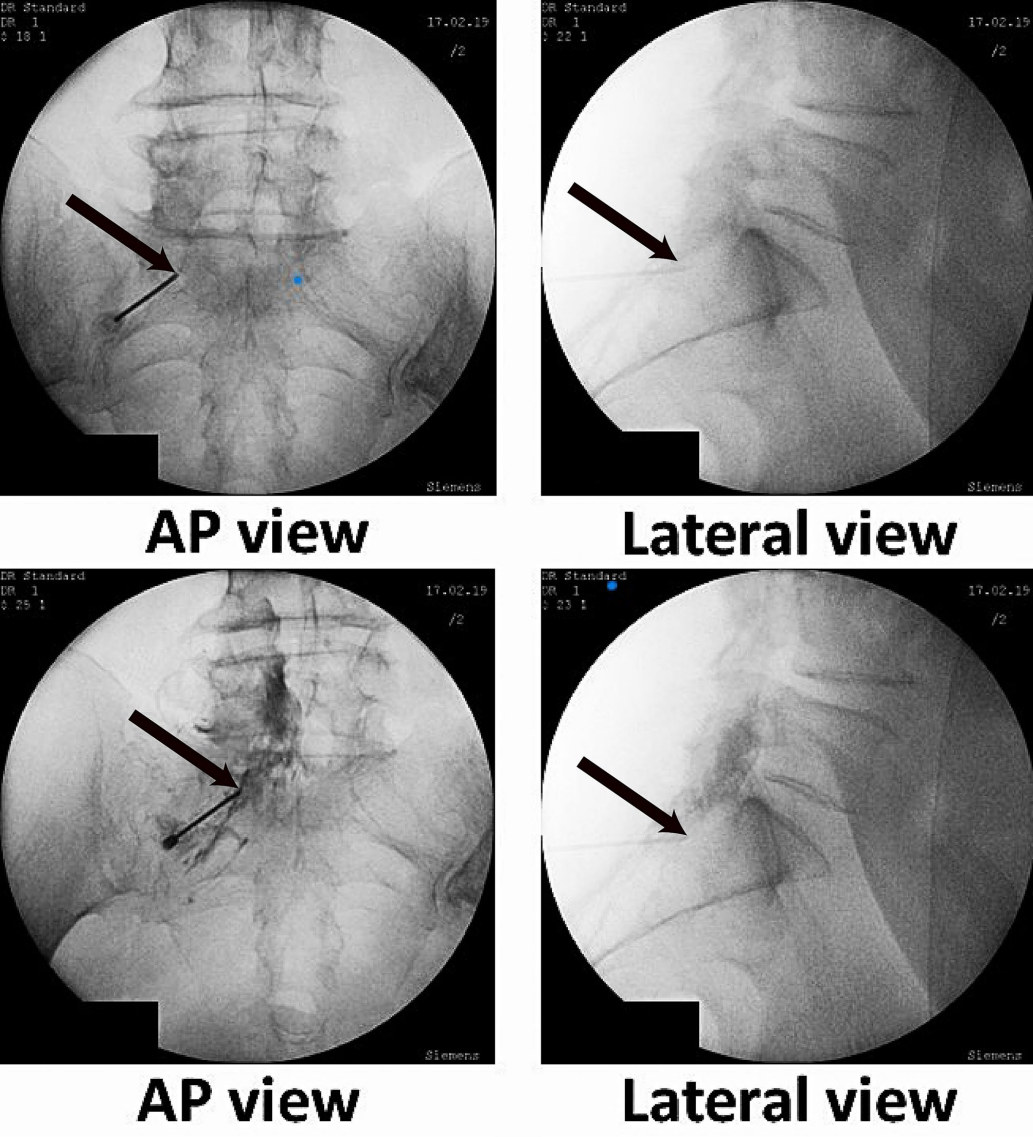
The needle is placed in epidural space through the left S1 transforaminal approach. Needle tips are shown with black arrows. Contrast medium can be seen outlining the S1 nerve root and spreading into the epidural space.

“Anterior” spread was defined if the contrast reached the posterior longitudinal ligament or touched the posterior wall of the vertebral body at the level of needle placement (Fig. 2). Due to the unavailability of dose–area product (DAP) or fluoroscopy time data, we were restricted to recording the total count of fluoroscopic images in both groups as a surrogate measure.

### Sample size calculation

Sample size was calculated using OpenEpi (V. 3.01, Open Source Epidemiologic Statistics for Public Health, www.OpenEpi.com), assuming a type 1 error of 0.05 and a statistical power of 0.8 to achieve a 30% difference between study groups. A sample size of 40 patients per group was deemed sufficient. Additionally, this sample size satisfies a non-inferiority design (α = 0.05, β = 0.8), assuming a non-inferiority margin of 0.1 and a predicted efficacy difference of 20%, as calculated by an online calculator (https://www2.ccrb.cuhk.edu.hk/stat/proportion/tspp_sup.htm).

### Randomization and blinding

We randomly assigned the patients to the TF or PIL groups using block randomization with a block size of 4. The randomization sequence was generated using the Microsoft Excel RAND function by an independent researcher. This allocation sequence was concealed from the principal investigator and the treating physician. The randomization list was generated for a total of 88 subjects to account for potential attrition.

### Procedures

All procedures were performed by the same pain fellowship-trained anesthesiologist with more than 20 years of experience, using the same C-arm fluoroscopy machine and radiology technologist. The vertebral level and the left or right sides were determined using clinical examination and diagnostic images.

**Fig. 3:**
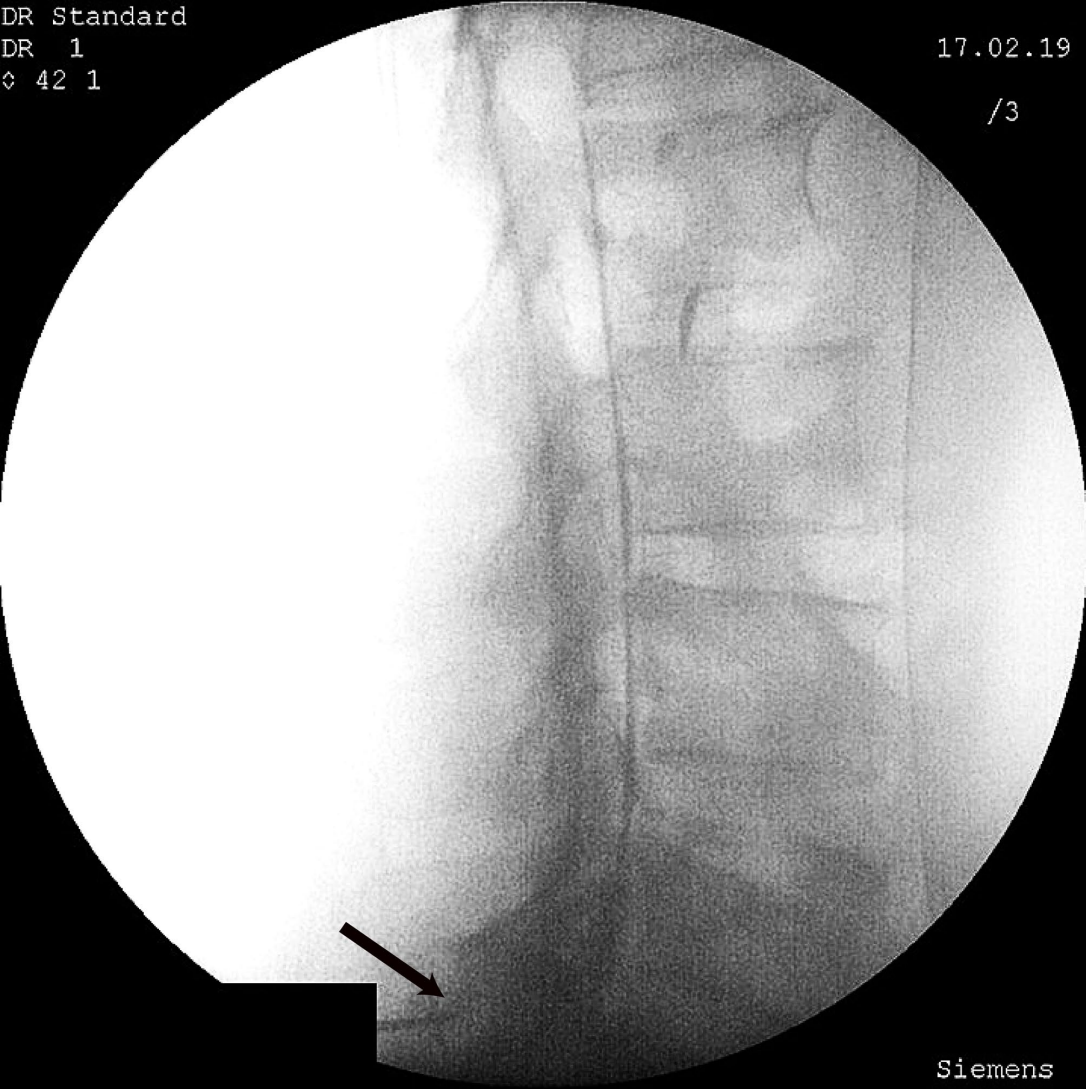
The needle is placed in L5-S1 parasagittal interlaminar epidural space. The black arrow indicates the needle tip. In lateral projection, the spread of contrast can be seen in anterior and posterior epidural spaces and multiple adjacent segments (railroad pattern). This image was taken to show contrast spread. The needle tip can be seen in the left bottom corner of the image.

In the PIL group, a 17 G Touhy needle (B Braun) was placed in the outermost aspect of the epidural space on the affected side, using the loss-of-resistance technique and under frequent Anteroposterior (AP) and Lateral C-arm fluoroscopy images (Fig. 3). In patients with a diagnosis of spinal stenosis undergoing a PIL approach, the injection site is modified to one level inferior to the maximal spinal stenosis level^22,23^.

**Fig. 4:**
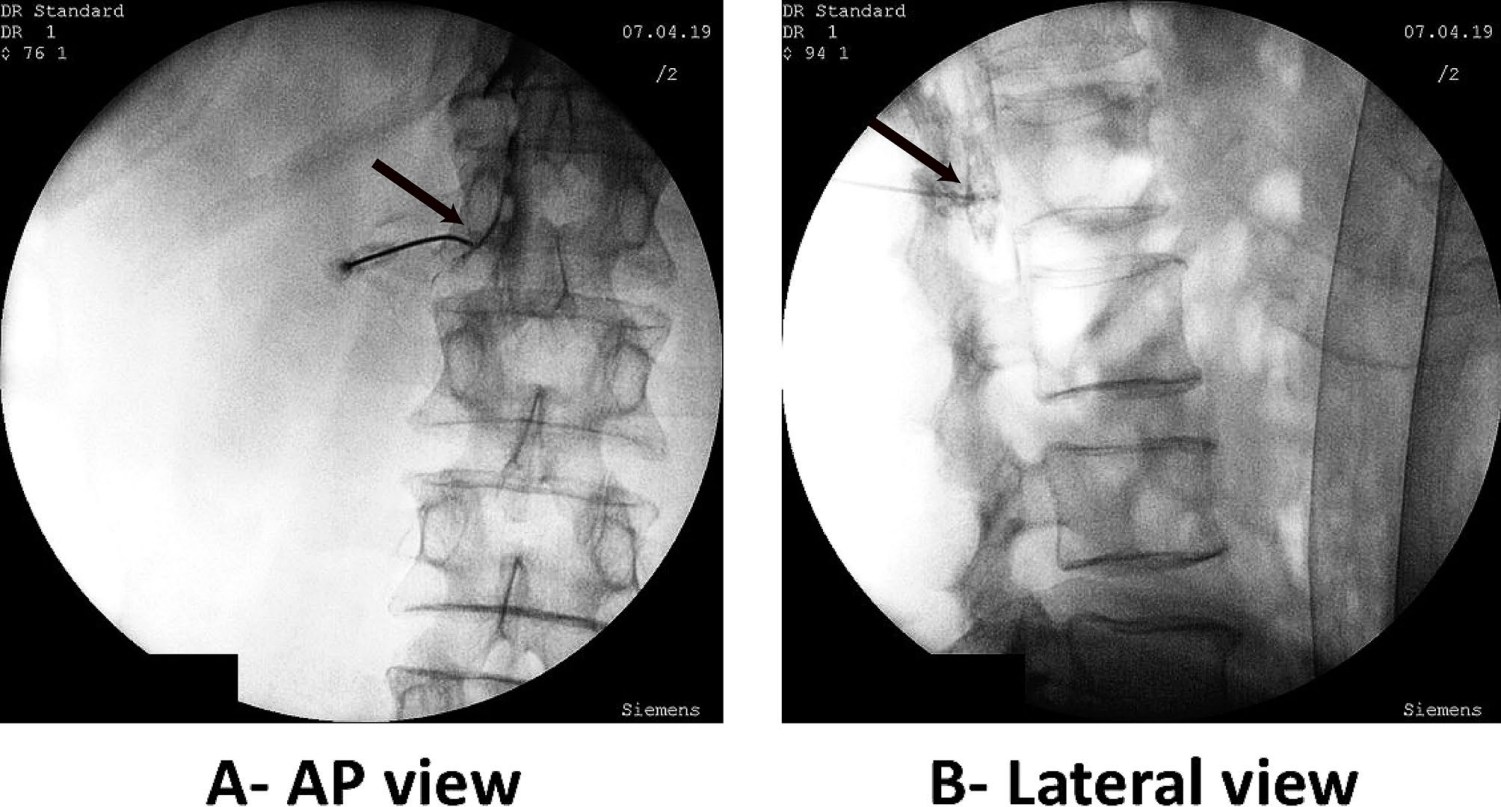
The needle is placed in epidural space through the left L1 transforaminal approach (**A**). The contrast spread in anterior and posterior epidural spaces and adjacent epidural segments can be seen in lateral projection (**B**). Needle tips are shown with black arrows.

In the TF group, under intermittent Oblique (tunnel views), AP, and Lateral fluoroscopy images, a 22-G blunt curved needle was placed in the affected foramen under the pedicle (Fig. 4).

In both groups, 5 ml of iohexol 240 was slowly injected into the epidural space to confirm needle placement and rule out the intravascular, subarachnoid, subdural, or intradiscal injections. Fluoroscopic images were carefully saved for later review of contrast spread in the epidural space. Then, we prepared 10 ml of a mixture consisting of 40 mg of methylprednisolone, 4 ml of normal saline, and 5 ml of 2% lidocaine^24^. This total volume was injected into the interlaminar epidural space. For the TF group, we prepared 3 ml of a mixture consisting of 40 mg methylprednisolone (1 ml), 1.5 ml of 2% lidocaine, and 0.5 ml of normal saline.

### Clinical assessment

Clinical outcomes were assessed by an independent observer who was strictly blinded to the patient’s group assignment. The observer recorded pain intensity and QOL pre-injection and at 2 weeks and at 1, 2, and 6 months after injection. To ensure valid assessments, the study employed a double-blind design regarding outcome evaluation.

#### Pain intensity

We evaluated and recorded the pain intensity with an 11-point Numeric Rating Scale (NRS). We asked for overall pain intensity in the preceding week before and at the moment of the patient’s visit to the pain clinic^25^. Pain relief was calculated as the percentage reduction in NRS score from baseline at each follow-up.

#### Quality of life (QOL)

We assessed the QOL using the Modified Oswestry Low Back Pain Questionnaire (MOLBPQ). This questionnaire has been locally validated and confirmed for the local population of this study^26^. It is a widely used tool for evaluating the impact of low back pain on an individual’s ability to perform daily activities. The questionnaire consists of 10 items that assess the degree of interference caused by low back pain in various aspects of life, including personal care, lifting, walking, sitting, standing, sleeping, sex life, social life, and traveling. Each item is scored on a scale from 0 to 5, with higher scores indicating greater disability. The scores for each item are then summed to yield a total score, which ranges from 0 to 50.

#### Satisfaction with treatment

A zero-to-10 self-report numerical scale was used to measure satisfaction with treatment in patients. The number 10 indicates the highest level of satisfaction, and the number zero means dissatisfaction with treatment.

**Global Perceived Effect (GPE)**: The Global Perceived Effect (GPE) is a method commonly used in both research and practice to evaluate patients’ assessment of their condition. GPE in patients with low back pain, as employed by Chang et al^27^., was assessed with a 7-point Likert scale: Very dissatisfied (score 1), dissatisfied (score 2), somewhat dissatisfied (score 3), undecided (score 4), somewhat satisfied (score 5), satisfied (score 6), and very satisfied (score 7).

Other studied variables included contrast diffusion, sex, age, weight, height, BMI, duration of pain, pathology, MRI findings, and EMG/NCV findings in TF-ESI and PIL-ESI technique groups^28,29^.

### Statistical analysis

The information was coded from the questionnaires and entered into SPSS.23 software (IBM Corp., Armonk, NY, USA). An intention-to-treat (ITT) approach was administered for the analysis of the data in this trial. Mean ± SD was used to report quantitative variables. Numbers and percentages were used for qualitative variables. Chi-square and Fisher’s exact tests were used to compare categorical data between groups. Quantitative data were analyzed for normal distribution by the Shapiro-Wilk test, and all were normally distributed; therefore, an independent samples t-test was used to compare these data between the two study groups. The significance level was considered less than 0.05.

## Results

A total of 40 patients were enrolled in each group. The demographic and pre-procedure data are shown in Table 1. There was no significant difference between the two groups in the demographic parameters and in the pre-procedure data. Consequently, the two groups were comparable in baseline characteristics (Table 1).

**Table 1:** Demographics and pre-procedure characteristics of patients in the PIL and TF groups.

Variable	PIL Group (*n* = 39)	TF Group (*n* = 40)	*P*-value
Age, years	55.69 ± 14.78	55.73 ± 12.97	0.992
Sex, male	12 (30.8)	15 (37.5)	0.528
Weight, kg	77.59 ± 11.88	78.98 ± 12.00	0.608
Height, cm	165.36 ± 10.57	166.65 ± 11.74	0.609
Body mass index, kg/m2	28.50 ± 4.53	28.61 ± 4.69	0.915
Duration of pain, month	38.89 ± 46.80	34.14 ± 42.87	0.643
MRI disc findings			
Protrusion	11 (28.2)	12 (30.0)	0.795
Bulging & protrusion	13 (33.3)	14 (35.0)	
Bulging	4 (10.3)	6 (15.0)	
Extrusion	11 (28.2)	8 (20.0)	
MRI diagnoses (Stenosis/Degeneration)			
Foraminal Stenosis & degenerative disk disease	2 (5.1)	0 (0)	0.631٭
Spinal stenosis	5 (12.8)	3 (7.5)	
Degenerative disk disease	1 (2.6)	2 (5.0)	
Foraminal stenosis	6 (15.4)	8 (20.0)	
Disc pathology without significant stenosis or degeneration	25 (64.1)	27 (67.5)	
EMG-NCV findings			
Normal	5 (12.8)	2 (5.0)	0.841٭
Right L3	1 (2.6)	1 (2.5)	
Left L4	0 (0)	1 (2.5)	
Left L5	2 (5.1)	2 (5.0)	
Right L5	1 (2.6)	1 (2.5)	
Bilateral L5	3 (7.7)	7 (17.5)	
Bilateral S1	4 (10.3)	4 (10.0)	
Bilateral L3-L4	2 (5.1)	0 (0)	
Right L4-L5	1 (2.6)	3 (7.5)	
Left L5-S1	3 (7.7)	6 (15.0)	
Right L5-S1	2 (5.1)	2 (5.0)	
Bilateral L5-S1	11 (28.2)	7 (17.5)	
Bilateral L3-L4-L5-S1	2 (5.1)	1 (2.5)	
Bilateral L4-L5-S1	1 (2.6)	2 (5.0)	
Right L5-Left S1	1 (2.6)	1 (2.5)	

٭ P-values were calculated using Fisher’s exact test. Values are presented as standard deviation (SD) or number (%), Abbreviations: PIL: parasagittal interlaminar, TF: transforaminal.

Based on the results of the independent t-test, there was no statistically significant difference in the mean pain intensity between the two treatment groups at baseline or at any follow-up interval over the six-month period. Between-group effect sizes were small across all time points (Cohen’s d range: 0.00 to 0.23). The 95% confidence intervals of mean differences crossed zero at all time points, indicating that both techniques demonstrate equivalent analgesic efficacy (Table 2).

**Table 2:** Comparison of pain intensity (NRS) between PIL and TF epidural steroid injection groups over time.

Pain intensity	PIL group (*n* = 39)	TF Group (*n* = 40)	Mean difference (95% CI)	Effect size (Cohen’s d)	*P*-value
Pre-injection					
At the moment	5.46 ± 1.94	5.80 ± 2.18	−0.34 (−1.25 to 0.57)	0.165	0.470
Overall	5.46 ± 2.08	5.73 ± 2.23	−0.27 (−1.22 to 0.68)	0.125	0.590
2 weeks					
At the moment	3.49 ± 1.80	3.95 ± 2.09	−0.46 (−1.32 to 0.40)	0.236	0.297
Overall	3.13 ± 1.88	3.50 ± 2.19	−0.37 (−1.27 to 0.53)	0.181	0.422
1 month					
At the moment	3.62 ± 2.03	3.63 ± 1.87	−0.01 (−0.87 to 0.85)	0.005	0.983
Overall	3.33 ± 1.89	3.23 ± 1.96	0.10 (−0.75 to 0.95)	0.052	0.804
2 months					
At the moment	3.69 ± 1.93	3.60 ± 1.94	0.09 (−0.76 to 0.94)	0.047	0.833
Overall	3.64 ± 2.03	3.50 ± 2.27	0.14 (−0.81 to 1.09)	0.065	0.772
6 months					
At the moment	3.67 ± 2.49	3.95 ± 2.53	−0.28 (−1.39 to 0.83)	0.112	0.618
Overall	3.23 ± 2.12	3.60 ± 2.50	−0.37 (−1.39 to 0.65)	0.159	0.484

Values are presented as standard deviation (SD). Abbreviations: PIL: parasagittal interlaminar, TF: transforaminal.

The mean pain relief (in percentage), satisfaction, and QoL of patients during the six-month period after the treatment had no statistically significant difference between the two study groups (Table 3).

**Table 3:** Comparison of pain relief, satisfaction with treatment, quality of life, and global perceived effect (GPE) between PIL and TF epidural steroid injections.

Variable	PIL Group (*n* = 39)	TF Group (*n* = 40)	*P*-value
Pain relief			
2 weeks	58.97 ± 24.92	57.13 ± 25.54	0.746
6 months	49.49 ± 26.79	46.25 ± 31.61	0.625
Satisfaction with treatment			
2 weeks	7.00 ± 2.36	6.78 ± 2.46	0.680
6 months	5.92 ± 3.09	5.38 ± 3.74	0.480
Quality of life			
2 weeks	47.00 ± 0.22	44.00 ± 0.21	0.533
6 months	44.00 ± 0.24	38.00 ± 0.20	0.265
GPE			
2 weeks	4.10 ± 0.96	4.08 ± 0.91	0.897
6 months	3.59 ± 1.18	3.40 ± 1.35	0.510

Values are presented as mean ± standard deviation (SD). Pain relief was calculated as the percentage reduction in each patient’s numeric rating scale pain score from baseline at each follow-up. Satisfaction was reported using a 0–10 numerical scale. Quality of life was recorded according to the Modified Oswestry Low Back Pain Questionnaire (MOLBPQ). GPE was assessed with a 7-point Likert scale. Abbreviations: GPE: global perceived effect, PIL: parasagittal interlaminar, TF: transforaminal.

Table 4 shows that the PIL group had a higher percentage of injections on the right side and in the L5-S1 level (56.4%). The TF group had a higher percentage of injections on the left side and at the L5 level (77.5%). The P-value of 0.651 indicates that there is no significant difference in the distribution of the sides between the two groups.

**Table 4:** Distribution of injection levels and sides for the PIL and TF epidural steroid injections.

	L2-L3	L3	L3-L4	L4	L4-L5	L5	L5-S1	S1	Right	Left
PIL, (%) *n* = 39	1 (2.6)	0 (0)	3 (7.7)	0 (0)	13 (33.3)	0 (0)	22 (56.4)	0 (0)	19 (48.7)	20 (51.3)
TF, (%) *n* = 40	0 (0)	1 (2.5)	0 (0)	6 (15)	0 (0)	31 (77.5)	0 (0)	2 (5)	21 (52.5)	19 (47.5)
Total (%) *n* = 79	1 (1.3)	1 (1.3)	3 (3.8)	6 (7.6)	13 (16.5)	31 (39.2)	20 (25.3)	3 (3.8)	40 (50.6)	39 (49.4)

Values are presented as number (%). P-value = 0.651 for the distribution of the sides between the two groups. Abbreviations: PIL: parasagittal interlaminar, TF: transforaminal.

Diffusion of the contrast in the anterior epidural space in all cases extended to more than two vertebral levels (Table 5). The comparison of contrast diffusion into the anterior epidural space between the two groups showed no statistically significant difference (*P* = 0.436). However, the caudal distribution of the contrast was greater in the PIL group than in the TF group. The mean number of fluoroscopic images taken in the PIL and TF groups was significantly different, with fewer imagesin the PIL group (Table 5). No complications occurred in this study.

**Table 5:** Number of vertebral levels of epidural contrast spread following IL and TF epidural steroid injection and number of fluoroscopic shots.

Spread of contrast	PIL Group (*n* = 39)	TF Group (*n* = 40)	*P*-value
Anterior epidural space	4.90 ± 2.03	4.51 ± 2.22	0.436
Cephalad spread	3.16 ± 1.64	2.78 ± 1.41	0.270
Anterior cephalad spread	2.5 ± 1.87	2.43 ± 1.55	0.853
Posterior cephalad spread	2.72 ± 1.52	2.18 ± 1.30	0.107
Caudal spread	2.70 ± 0.81	2.10 ± 0.78	0.001^*^
Anterior caudal spread	2.36 ± 0.74	1.85 ± 0.63	0.002 ^*^
Posterior caudal spread	2.67 ± 0.99	1.90 ± 0.79	0.001 ^*^
Number of fluoroscopic shots	12.05 ± 6.66	23.25 ± 10.73	< 0.001^*^

Values are presented as mean ± standard deviation (SD). Abbreviations: PIL: parasagittal interlaminar, TF: transforaminal.

^*^ Statistically significant with *p* < 0.05.

## Discussion

This study demonstrated that clinical outcomes did not differ significantly between the high-volume PIL and target-specific TF groups. Both techniques yielded comparable ventral contrast spread, pain relief, and patient satisfaction across all follow-up time points up to six months. However, the number of fluoroscopic images required in the TF group was significantly higher than in the PIL group.

Regarding clinical relevance, reductions in NRS scores exceeded the minimal clinically important difference (MCID) of 2 points at most follow-up time points in both groups. To avoid losing clinical data from patients with moderate relief (such as 40–50%) due to strict cutoffs, we analyzed pain relief as a continuous variable rather than classifying patients into “responders” and “non-responders”. As shown in Table 3, the mean percentage of pain relief at the 2-week follow-up was 58.97% in the PIL group and 57.13% in the TF group, exceeding the 50% threshold. This suggests that the interventions were clinically effective for the majority of the study population at two weeks. Furthermore, the 6-month data showed sustained mean relief near 50% (49.49% vs. 46.25%), highlighting the long-term durability of both approaches.

Our findings are consistent with previous studies comparing the efficacy of PIL and TF-ESI for lumbosacral radicular pain. In the present study, pain intensity decreased significantly in both treatment groups over six months. This finding aligns with the results of Beyaz et al., who evaluated these two approaches in patients with chronic low back pain over 12 months. That study demonstrated a significant reduction in mean pain scores in both groups, with 85.1% of patients reporting satisfaction^30^. Similarly, another study observed significant pain relief in both IL- and TF-ESI cohorts with no statistically significant difference between them^31^. Although no adverse effects were observed in our study following ESI in either group, existing literature indicates the TF approach is associated with a higher rate of complications compared to IL-ESI^32^.

Our study suggests that the PIL approach is associated with more extensive caudal contrast spread compared to the TF approach. This difference in contrast distribution may influence the comparative efficacy of the two modalities. Regarding the clinical significance of distribution, Lutz and Wisneski evaluated 50 patients with lumbar radiculopathy following intervertebral disc herniation; they showed that clinical improvement following TF-ESI correlated with increased ventral contrast spread^33^. In our cohort, ventral contrast spread within the epidural space extended beyond two vertebral levels in all cases, with no statistically significant difference observed between the PIL and TF groups (*p* = 0.138). Conversely, a systematic review and meta-analysis showed higher rates of ventral contrast spread in the PIL group compared to the TF approach. The authors also noted that the PIL method yielded more effective pain relief^13^.

Regarding procedural safety, the PIL procedure appears to be less time-consuming and requires less expertise. A systematic review and meta-analysis confirmed that PIL showed benefits in terms of lower mean fluoroscopy time, less radiation exposure, and no reported adverse events or intravascular spread^13^. In contrast, the TF technique is associated with prolonged procedural duration and increased radiation doses, particularly in complex cases such as lumbar spinal stenosis^34^. Furthermore, fluoroscopically guided lumbar TF epidural injections also result in higher physician radiation exposure compared to CT-guided procedures^35^. Because of these elevated risks, our findings emphasize the critical need to optimize fluoroscopy during pain management procedures. Adherence to the ALARA (As Low As Reasonably Achievable) principle is essential^36^. Several strategies can be employed to minimize radiation exposure. For example, utilizing pulsed fluoroscopy is advised to lower the total time and number of images compared to continuous fluoroscopy, thereby limiting the dose for both the physician and patient. Additionally, collimation can narrow the X-ray field^37,38^. Moreover, the use of protective gear and shielding can further mitigate exposure^38^. Finally, maximizing the distance between the physician and the X-ray source is critical, as radiation is inversely proportional to the square of the distance from the source^36^.

A distinct advantage of TF-ESI is that if the pressure or inflammation is limited to a specific foramen, then the drug can be administered in very close proximity to the lesion. If the number of the affected roots is limited, the TF-ESI will need less medication volume, potentially reducing the risk of side effects of the injectate.

Regarding medication choice, particulate steroids were utilized in this study. Existing literature is conflicting regarding the optimal steroid for pain relief in TF injection^39–43^. While nonparticulate steroids, such as dexamethasone, are often recommended to minimize thromboembolic risk, particulate steroids can be used in lumbar injections with caution, based on availability^44^ and evidence suggesting superior long-term benefits for pain and disability in specific contexts. Future studies should directly compare the safety and efficacy of particulate versus nonparticulate agents in both TF and PIL approaches.

Previous studies have shown that MRI findings may predict the response to TF-ESI. While disc morphology alone (bulging, protrusion, or extrusion), spinal stenosis, and lumbar disc herniation accompanied by degenerative changes were not associated with TF-ESI outcomes, better pain relief has been reported in patients with lower-grade compression and non-subarticular disc herniations (central, foraminal, or extraforaminal)^45–47^. In our study, MRI characteristics did not differ significantly between the PIL and TF groups, and both groups showed a comparable distribution of MRI findings, such as stenosis, disc degeneration, protrusion, bulging, and extrusion (Table 1, *p* > 0.05 for all morphological categories). We did not perform subgroup analysis because stratifying our limited sample size would reduce statistical power. Future studies with larger sample sizes should evaluate whether specific pathologies benefit more from a particular technique. Similarly, widespread EMG abnormalities have been associated with worse outcomes in prior research. In our study, EMG findings were similar between groups (59% bilateral involvement in PIL vs. 50% in TF), suggesting comparable baseline severity^46^.

The injected volumes in this study differed between groups (10 ml for PIL vs. 3 ml for TF). This difference was necessitated by the anatomical requirements of the two approaches. In the TF technique, the needle is placed close to the nerve root; therefore, a smaller volume is sufficient to reach the target while minimizing the risk of excessive foraminal pressure^48^. In contrast, the PIL approach delivers the injection into the dorsal epidural space. Consequently, a larger amount (10 ml) is required to ensure adequate craniocaudal spread to the anterior nerve roots. This dosage distinction is consistent with previous studies^49,50^. Future protocols should aim to standardize injectate amounts or perform sub-analyses to distinguish the influence of volume from that of the injection site on clinical outcomes.

Regarding contrast administration, while iohexol is typically administered in volumes of 0.5–3 mL for TF injections^51^, we used 5 ml to allow clear visualization of the ventral epidural spread. Prior studies suggest that larger contrast volumes may improve visualization and enhance drug delivery to the ventral epidural space^52–54^. However, using higher volumes may lead to reduced targeting accuracy, dilution of medications, increased injection pressures, and potentially a higher risk of complications. Future studies should investigate the optimal contrast volume in each patient’s anatomy^51^.

Although we used the total number of fluoroscopic images to compare radiation exposure, this variable serves as a surrogate rather than a direct measure. The dose should be calculated in a radiation exposure unit (e.g., mREM, Gy, Sv). However, when fluoroscopic equipment does not automatically record KAP/DAP or enable equivalent dose calculation, alternative metrics such as fluoroscopy time and the number of fluorographic images may be used as surrogates, as utilized in the present study. Future investigations should prioritize objective radiation quantification, preferably via DAP or exposure duration, to enhance methodological validity.

In this study, we used anteroposterior (AP) and lateral fluoroscopic views in the PIL approach to visualize needle placement and did not employ the contralateral oblique (CLO) view. In the CLO technique, the C-arm is rotated 45° toward the opposite side of the needle entry. As shown by Gill et al., a 45° CLO view provided grade-1 needle-tip visualization in 100% of cases and accurately localized the tip on the ventral interlaminar line (VILL) in 79%, whereas lateral fluoroscopy achieved grade-1 visualization in only 65% and VILL localization in 35%^55^.. These capabilities reduce the need for needle repositioning and repeat imaging and lower complications. However, lateral views remain superior for monitoring anterior contrast spread^56^.

An important strength of this study is the long-term follow-up of six months. In addition to evaluating ventral epidural contrast spread, we assessed pain intensity, quality of life, and patient satisfaction across both groups. Nevertheless, expanding the follow-up period beyond six months in future studies could strengthen conclusions about long-term efficacy.

### Clinical implications

The comparable clinical efficacy and anterior epidural contrast spread of high‑volume PIL‑ESI and TF‑ESI support the use of the PIL approach as an alternative to the TF route, particularly in challenging scenarios such as severe foraminal stenosis, prior surgery, distorted anatomy, or vascular risk. In such patients, using PIL-ESI instead of TF can yield similar pain relief, function, and quality of life improvement. Furthermore, the lower number of fluoroscopic images in the PIL‑ESI technique, serving as a surrogate for reduced radiation exposure, may enhance procedural safety for both patients and staff and could be prioritized for individuals requiring repeated injections.

## Limitations

1. Population heterogeneity: Our study included patients with a broad range of lumbar pathologies, which may have influenced treatment response and could have impacted the overall results.
2. Radiation Metrics: We relied on the number of fluoroscopic images as a surrogate for radiation exposure because DAP or dose data were unavailable. Additionally, the lack of standardized collimation protocols may have influenced the total radiation burden.
3. Steroid Selection: We utilized particulate steroids; while effective, they carry a different safety profile compared to nonparticulate agents regarding thromboembolic risk.
4. Systemic effects: We did not measure systemic steroid suppression, such as cortisol levels or other assessments of hypothalamic-pituitary-adrenal (HPA) axis function.
5. Contrast Volume: The use of higher contrast volumes in the TF group, while intended to improve visualization, carries a theoretical risk of medication dilution or increased pressure.
6. Subjective variability: Pain intensity measurements yielded relatively large and overlapping standard deviations, which may show variability in subjective reporting. We attempted to mitigate this limitation by using multiple outcome measures at different time points, blinded assessment, and objective imaging-based endpoints.

## **Conclusion**

The clinical outcomes, including pain intensity, pain relief, patient satisfaction, and quality of life, were not significantly different between the high-volume PIL and TF epidural steroid injection techniques over a six-month follow-up period. The spread of the contrast in the anterior EP space was comparable in both groups. However, the fluoroscopic images in TF-ESI were significantly more numerous than in high-volume PIL-ESI. We would recommend that further studies be performed with larger populations and over a longer period across various pathologies to elucidate the advantages of these two ESI techniques. They should also perform accurate radiation assessments for both techniques.

## Data Availability

The original contributions presented in the study are included in the article; further inquiries can be directed to the corresponding author.
